# Psycho-social predictors of motivation for treatment in patients with mental disorders: the role of adverse childhood experiences and internalized stigma.

**DOI:** 10.1192/j.eurpsy.2024.1490

**Published:** 2024-08-27

**Authors:** N. Lutova, E. Gerasimchuk, M. Sorokin, M. Bocharova

**Affiliations:** V.M. Bekhterev National Medical Research Centre for Psychiatry and Neurology, St.Petersburg, Russian Federation

## Abstract

**Introduction:**

Motivation for treatment is an important socio-psychological characteristic of patients, which is subject to the joint influence of various factors, each of which may require specific rehabilitation interventions.

**Objectives:**

To analyze and evaluate the cumulative influence of adverse childhood experiences (ACE), internal stigma, social characteristics on the intensity of treatment motivation in patients with mental disorders.

**Methods:**

102 patients with mental disorders were examined using Adverse Childhood Experience Questionnaire (ACEQ), Russian-language validated Internalized Stigma of Mental Illness (ISMI) scale and Treatment Motivation Assessment Questionnaire (TMAQ).

**Results:**

As a result of regression analysis (table 1), a model was obtained that predicted an increase in the chances of high patient’s motivation for treatment with an increase in the total score of ACEs (ACEQ total score) and with higher education. The overall severity of internal stigma (ISMI total score) did not show a significant effect on the chances of developing intense motivation in patients.

**Table 1.** Model of logistic regression analysis of educational, ACE total score and ISMI total score with the severity of motivation for treatment.

Predictor	B	SE	p	Exp (B)	95% confidence interval for EXP(B)
Secondary education	1,120	0,699	0,109	3,065	0,778-12,074
Higher education	1,972	0,775	,011	7,189	1,574-32,834
ISMI total score	-0,435	0,773	0,574	0,647	0,142-2,946
ACEQ total score	0,346	0,147	0,019	1,414	1,060-1,886

After post data analysis (table 2), a cut-off point was established for the ACEQ total score of 4 points, corresponding to an increased chances of high patient’s treatment motivation.

**Table 2.** Results of the test ROC analysis for ACEQ total score and the severity of motivation for treatment.

Cutpoint	Sensitivity (%)	Specificity (%)	Youden’s index	AUC
3	64.71%	53.57%	0.183	0.689
4	50%	75%	0.250	0.689
5	38.24%	85.71%	0.239	0.689

**Conclusions:**

ACEs may likely be a source of posttraumatic growth in adulthood in patients with mental illness specially if their count amounts to 4 or more variants. The role of social and psychological characteristics of patients with mental disorders in the psychology of the treatment process should be considered systemically, rather than discretely.

**Disclosure of Interest:**

None Declared

